# Poly-IgA immune complex as a monitoring biomarker of response to telitacicept in IgA nephropathy

**DOI:** 10.3389/fimmu.2026.1751549

**Published:** 2026-05-29

**Authors:** Rao Fu, Jingyang Gao, Yu Zhang, Zhe He, Xuanyi Du

**Affiliations:** Department of Nephrology, Second Affiliated Hospital of Harbin Medical University, Harbin, China

**Keywords:** APRIL, BAFF, biomarker, IgA nephropathy, Poly-IgA, telitacicept

## Abstract

**Background:**

Immunoglobulin A nephropathy (IgAN) is a common immune-mediated kidney disease. Its pathogenesis involves dysregulated B cell activation, excessive production of galactose-deficient immunoglobulin A1 (Gd-IgA1), and formation of IgA immune complex, which collectively drive glomerular injury. Telitacicept, a fusion protein that blocks B cell activation pathways, may counteract these mechanisms, but observational data and reliable biomarkers of therapeutic response remain limited.

**Objective:**

This study aimed to evaluate the clinical efficacy and safety of telitacicept and to determine whether changes in circulating B cell activating factor (BAFF), a proliferation-inducing ligand (APRIL), and Poly-IgA immune complex (Poly-IgA) reflect treatment response.

**Methods:**

In this single-center retrospective cohort study, 38 adult patients with IgAN received telitacicept combined with low-dose glucocorticoids for up to 9 months. Clinical outcomes, renal function, and safety events were monitored longitudinally, and serum biomarkers were analyzed to assess associations with remission.

**Results:**

Telitacicept treatment resulted in a substantial and sustained reduction in proteinuria while maintaining stable renal function, with over half of patients achieving complete or partial remission at 3 months. All three biomarkers reduced with treatment, and Poly-IgA showed the strongest correlation with clinical remission and the best ability to distinguish remission from non-remission.

**Conclusion:**

Telitacicept is an effective and well tolerated drug in the treatment of IgAN, with immunomodulatory effects consistent with suppression of pathogenic B cell activity. The lower level of Poly-IgA during treatment is significantly associated with clinical remission.It may serve as a promising biomarker for monitoring treatment response in the future, supporting its potential use in guiding individualized therapy for patients with IgAN.

## 1 Introduction

Immunoglobulin A nephropathy (IgAN), first described by Berger and Hinglais in 1968 (1), is now recognized as the most common primary glomerular disease worldwide. Its epidemiological characteristics have become increasingly well defined, revealing marked geographic and ethnic variation. Studies indicate that IgAN accounts for more than 50% of biopsy-confirmed primary glomerulonephritis in many Asia-Pacific regions, including China and Japan, as well as several Western European countries, whereas its incidence is notably lower in Africa and India (2). Importantly, approximately 25%–50% of individuals with primary IgAN progress to end-stage kidney disease (ESKD) within 20–30 years of follow-up (3), highlighting the significant and long-term clinical burden posed by this disorder.

According to the Kidney Disease: Improving Global Outcomes (KDIGO) 2025 guideline, the management of IgAN requires a dual and concurrent therapeutic strategy. This approach involves both mitigating the general consequences of nephron loss and targeting disease-specific pathogenic pathways. Foundational supportive care should be optimized through maximally tolerated renin–angiotensin system inhibition, use of sodium–glucose cotransporter-2 inhibitors for cardiorenal protection, and stringent blood pressure control, with a target of ≤120/70 mmHg. Simultaneously, treatment targeting the immunopathogenesis of IgAN should be implemented; targeted-release budesonide is recommended to reduce the production of galactose-deficient IgA1 and subsequent formation of IgA immune complex. The overarching treatment objective is to decrease proteinuria to <0.5 g/day (ideally <0.3 g/day) and to slow the rate of kidney function decline (4).

Currently, the pathogenesis of IgAN is described by the “four-hit” hypothesis (5). Circulating galactose-deficient IgA1 (Gd-IgA1) derived from mucosal immune responses is recognized by circulating IgG or IgA autoantibodies, ultimately forming pathogenic Poly-IgA immune complex (Poly-IgA). These complexes are the main source of IgA1 deposition in the glomerular mesangium, and their components include not only Poly-IgA but also IgG, IgM, and complement components. Studies have shown that the receptor CD89 on the surface of myeloid cells has a higher affinity for Poly-IgA than for monomeric IgA, enabling phagocytes to selectively capture Poly-IgA (6). These complexes then deposit in the glomerular mesangium, activate mesangial cells, and trigger glomerular injury (5, 7, 8). Therefore, therapeutic strategies aimed at reducing or preventing the formation of IgA immune complex have become the core focus of IgAN management.

Gd-IgA1 originates from aberrant B lymphocytes and plasma cells in mucosa-associated lymphoid tissue (9). B cell maturation and IgA class switching occur through both T cell–dependent and T cell–independent pathways. The T cell–independent pathway is driven by B cell activating factor (BAFF) and a proliferation-inducing ligand (APRIL), which are secreted by dendritic cells and macrophages (10, 11). These cytokines signal through receptors such as BAFF-R, TACI, and BCMA, with BAFF mainly supporting early B cell maturation and APRIL promoting later differentiation and plasma cell survival (11). The T cell–dependent pathway involves interactions between follicular helper T cells and B cells within germinal centers, a process further modulated by the coordinated activity of BAFF and APRIL (10).

Telitacicept is an innovative biologic agent engineered as a recombinant fusion protein combining the extracellular domain of the transmembrane activator and calcium-modulator cyclophilin ligand interactor (TACI) with the Fc portion of human IgG. By simultaneously blocking signaling through BAFF and APRIL, telitacicept suppresses abnormal B cell activation, limits plasma cell differentiation, and reduces the production of Gd-IgA1 and the formation of Poly-IgA (12). Emerging clinical data indicate that telitacicept can significantly lower proteinuria and stabilize kidney function (13), highlighting its potential as a targeted therapeutic option for IgAN. However, reliable serum biomarkers capable of objectively reflecting treatment response remain limited.

Therefore, this study was designed to systematically evaluate the efficacy and safety of telitacicept, characterize its effects on circulating biomarker profiles, and examine the relationships between these biomarkers and clinical remission, with the goal of monitoring treatment and supporting individualized therapy.

## 2 Methods

### 2.1 Study participants

This single-center retrospective study enrolled 38 patients diagnosed with IgAN at the Department of Nephrology, Second Affiliated Hospital of Harbin Medical University, between January 2024 and December 2025. Eligible participants were required to be aged ≥ 18 years, have biopsy-confirmed primary IgAN, present with 24-hour urinary protein excretion of at least 0.75 g(despite maximally tolerated doses of renin-angiotensin system inhibitors for at least 3 months), and have an estimated glomerular filtration rate (eGFR) greater than 30 ml/min/1.73 m², which was calculated using the 2009 CKD-EPI creatinine equation (CKD-EPIcr-2009) (23), and provide written informed consent. Exclusion criteria included secondary IgAN such as that associated with Henoch–Schönlein purpura, systemic lupus erythematosus, ankylosing spondylitis, liver cirrhosis, or infection, severe cardiovascular, cerebrovascular, malignant hypertension, or other systemic diseases, absence of baseline or follow-up data, treatment duration shorter than 9 months, and pregnancy or lactation. Healthy control subjects (n = 8) were selected from the health checkup center of the same hospital during the same study period; inclusion criteria were normal renal function (eGFR ≥ 90 mL/min/1.73 m²), urinalysis negative for protein and occult blood, no history of kidney or autoimmune disease (including IgAN), and no immunosuppressive therapy within 3 months. Serum samples from healthy controls were used to establish reference levels of the studied biomarkers and to compare with IgAN patients. The study protocol was approved by the Institutional Review Board of the Second Affiliated Hospital of Harbin Medical University (Approval No. YJSKY2024-086).

### 2.2 Experimental design

#### 2.2.1 Treatment protocol

All enrolled patients received combination therapy consisting of subcutaneous telitacicept 160 mg administered once weekly at rotating injection sites (upper arm, abdomen, or upper thigh), together with low-dose glucocorticoids (specifically methylprednisolone) at 0.2–0.4 mg/kg per day, not exceeding a maximum daily dose of 24 mg. In addition, all patients continued standard-of-care therapy with angiotensin-converting enzyme inhibitors or angiotensin II receptor blockers, titrated to the highest tolerated dose. The total treatment duration for each patient was 9 months.

#### 2.2.2 Data collection

Clinical data were collected at baseline and at 1, 3, 6, and 9 months after treatment initiation, including general demographic and clinical characteristics as well as laboratory results. Adverse events were documented throughout the study period. General information included sex, age, and comorbidities (e.g., hypertension, diabetes). Laboratory assessments consisted of 24-hour urinary protein excretion, urinary red blood cell (RBC) count, hemoglobin (Hgb), serum albumin (ALB), estimated glomerular filtration rate (eGFR) calculated using the CKD-EPI equation, and renal pathology classified according to the Oxford MEST−C classification (M: mesangial hypercellularity; E: endocapillary hypercellularity; S: segmental glomerulosclerosis; T: tubular atrophy/interstitial fibrosis; C: crescents) for IgAN. The results of urine sediment microscopy are all reported as the number per high-power field (cells/HPF). Peripheral blood samples (5 ml) were obtained from 16 patients with IgAN at baseline and again at 1, 2, 3, and 6 months of treatment. Samples were centrifuged at 3000 rpm for 5–10 minutes, and the resulting supernatant was aliquoted into cryovials and stored at –80 °C. Blood samples collected from healthy controls at our hospital’s physical examination center underwent identical processing. Serum concentrations of BAFF, APRIL, and Poly-IgA were quantified using commercially available enzyme-linked immunosorbent assay (ELISA) kits.

#### 2.2.3 Measurement of BAFF and APRIL

Serum concentrations of BAFF and APRIL were measured using commercially available ELISA kits (Jiangsu Enzyme Immunity Industrial Co., Ltd., Jiangsu, China; BAFF kit no. MM-12518H2; APRIL kit no. MM-51464H2) based on a dual-antibody, one-step sandwich enzyme-linked immunosorbent assay. All assays were performed strictly according to the manufacturer’s instructions, and each sample was analyzed in accordance with standardized laboratory quality control procedures.

#### 2.2.4 Measurement of Poly-IgA

Serum Poly-IgA levels were quantified using a commercially available ELISA kit (Lujing Biotech Co., Ltd., Shenzhen, China; kit no. NephroPlex_96T). HQP001 protein was used as the capture molecular probe for specific detection of Poly-IgA. Serum samples were diluted 1:1000 with the provided sample dilution buffer prior to analysis, and all subsequent assay procedures were performed in strict accordance with the manufacturer’s instructions.

#### 2.2.5 Study endpoints

The primary endpoint was the absolute and percentage change from baseline in 24-hour urinary protein excretion over the course of follow-up. Secondary endpoints included the absolute and percentage changes from baseline in eGFR, urinary RBC count, and serum concentrations of Poly-IgA, BAFF, and APRIL. Safety endpoints consisted of the incidence and type of adverse events (AEs), including injection site reactions, respiratory or urinary tract infections, hypotension, gastrointestinal discomfort, arthralgia, impaired glucose tolerance, abnormal liver function, and hyperkalemia. Serious adverse events (SAEs) were defined as all-cause mortality or other life-threatening events requiring hospitalization. Additionally, clinical remission outcomes relative to baseline, including complete remission (CR), partial remission (PR), and no remission (NR), were evaluated.

#### 2.2.6 Efficacy evaluation

Complete remission (CR) was defined as 24-hour proteinuria ≤ 0.3 g with stable renal function, indicated by an eGFR decline of no more than 30%, while partial remission (PR) was defined as a reduction of more than 50% in 24-hour proteinuria accompanied by stable renal function but not meeting the criteria for CR, and patients who failed to achieve CR or PR were classified as having no remission (NR); the overall response rate was calculated as the proportion of patients achieving either CR or PR divided by the total number of treated patients, expressed as a percentage.

### 2.3 Statistical analysis

Statistical analyses were performed using SPSS version 27.0 (IBM, Armonk, NY, USA). The Shapiro–Wilk test was applied to evaluate the normality of continuous variables. Normally distributed data were presented as mean ± standard deviation (SD) and compared between groups using the independent-samples t-test, while within-group comparisons were performed using paired t-tests. Non-normally distributed data were reported as median with interquartile range and analyzed using the Wilcoxon rank-sum test for between-group comparisons and the Wilcoxon signed-rank test for within-group analyses. Categorical variables were expressed as counts and percentages and compared using the chi-square test or Fisher’s exact test, as appropriate. Time-to-event outcomes, including time to complete remission and overall response, were assessed using Kaplan–Meier survival analysis. For variables with statistical significance in univariate analysis, multivariate COX proportional hazards regression models were used to analyze the independent prognostic value of each variable. Correlations between biomarkers and proteinuria were examined using Pearson or Spearman correlation coefficients according to data distribution. The performance of BAFF, APRIL, and Poly-IgA in assessing clinical response was evaluated using receiver operating characteristic (ROC) curve analysis and corresponding area under the curve (AUC) values. Due to the limited number of patients with available biomarker samples and the inconsistent timing of blood collection during follow-up, a formal longitudinal analysis at fixed time points could not be performed. Therefore, we conducted an exploratory cross-sectional analysis to evaluate the association between Poly-IgA levels measured at any time during treatment and the overall remission status assessed at the same time point (i.e., concurrent remission). This approach aims to make full use of existing data to generate hypotheses.A P-value < 0.05 was considered statistically significant.

## 3 Results

### 3.1 Baseline characteristics

**Figure 1:**
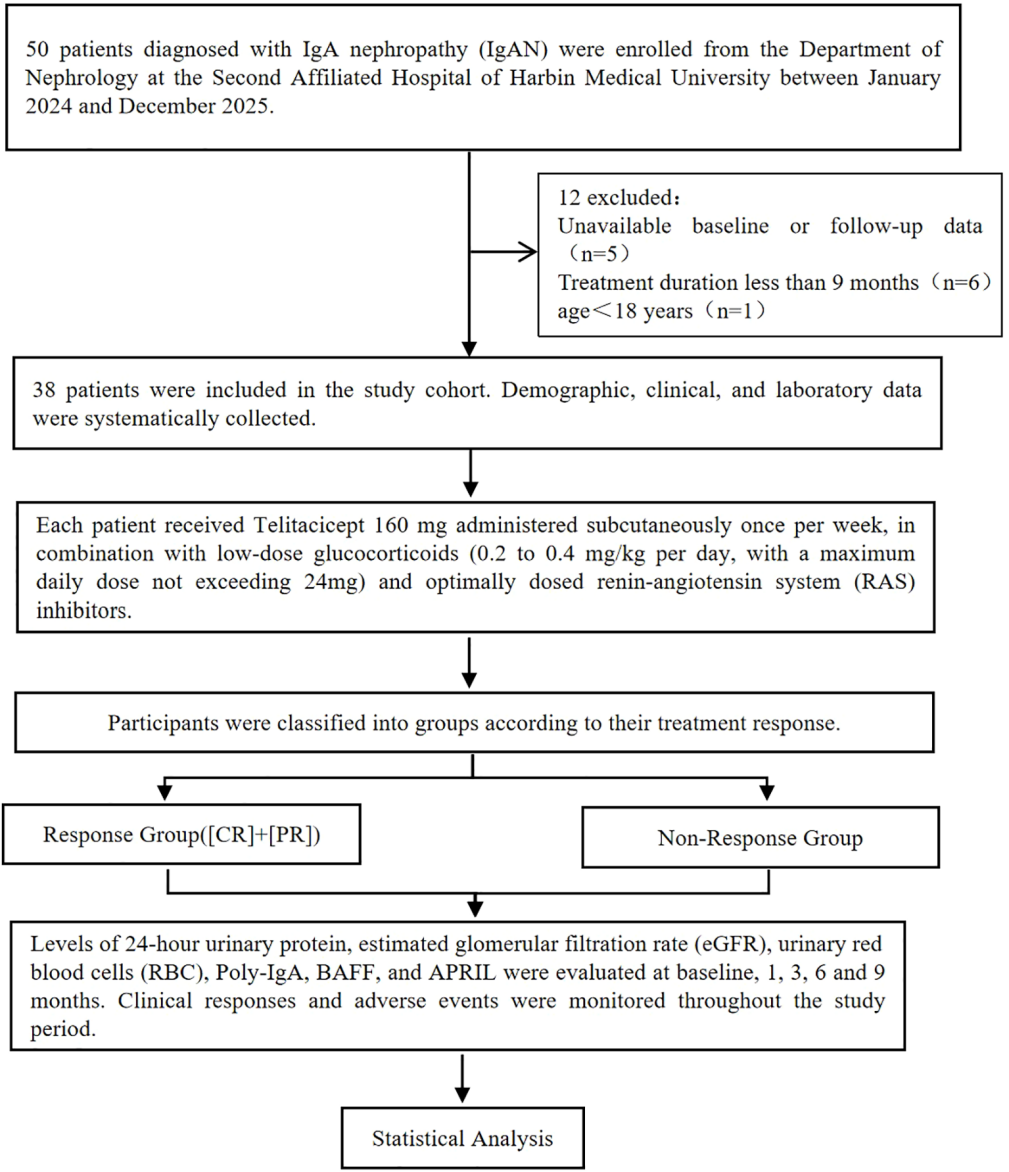
Technical roadmap.

A total of 38 eligible patients with IgAN were included in this study (**Figure 1**). The mean age of the patients was 38.37 ± 13.04 years, and 42.1% of the cohort were female. Hypertension and diabetes were present in 42.1% and 2.6% of patients, respectively. The median 24-hour urinary protein excretion was 1.75 g/day (interquartile range: 1.16–3.19), the mean eGFR was 83.69 ± 32.41 ml/min/1.73 m², and the median urinary RBC count was 22.00 cells/high-power field (interquartile range: 5.75–50.00). Based on the Oxford MEST-C classification, 86.8% of patients were M1, 28.9% were E1, 52.6% were S1, 76.3% were T1+T2, and 34.2% were C1+C2. Among the 16 patients who underwent serum biomarker assessment, the mean concentrations of BAFF, APRIL, and Poly-IgA were 270.48 ± 94.18 pg/ml, 26.40 ± 5.33 ng/ml, and 86.36 ± 44.49 µg/ml, respectively (**Tables 1A, B**).

**Table 1A:** Demographic, clinical characteristics and pathological classification.

Baseline characteristics of patients with IgAN treated with telitacicept (n=38)	
Age, mean(SD), yr	38.37(13.04)
Female sex, n (%)	16(42.1)
Hypertension, n(%)	16(42.1)
Systolic blood pressure, mean(SD), mmHg	126.16(18.70)
Diastolic blood pressure, mean(SD), mmHg	83.63(13.78)
Diabetes, n(%)	1(2.6)
Oxford classification, n	
M0/1	5/33
E0/1	27/11
S0/1	18/20
T0/1/2	9/28/1
C0/1/2	25/11/2

SD, standard deviation; M, mesangial hypercellularity; E, endocapillary hypercellularity; S, segmental glomerulosclerosis; T, tubular atrophy/interstitial fibrosis; C, crescents.

### 3.2 Overall efficacy evaluation

#### 3.2.1 Clinical remission

**Figure 2:**
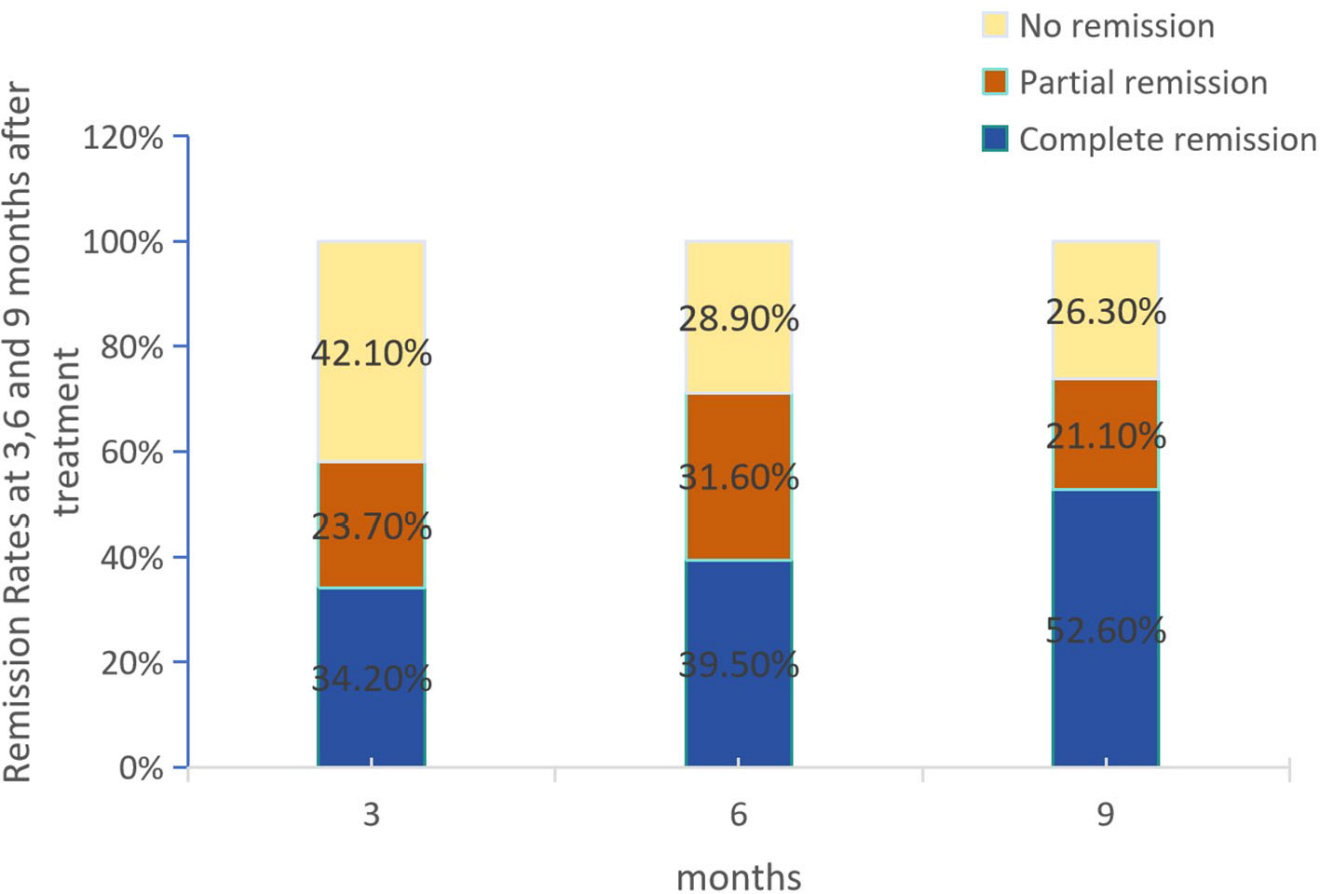
Remission rates at 3,6 and 9 months after treatment. Light Yellow: No remission; Orange: Partial remission; Dark Blue: Complete remission.

At 6 months of telitacicept treatment, 15 patients (39.5%) achieved CR and 12 patients (31.6%) achieved PR, resulting in an overall response rate of 71.1% (27 patients). At 9 months, 20 patients (52.6%) achieved CR and 8 patients (21.1%) achieved PR, yielding an overall response rate of 73.7% (28 patients) (**Figure 2**, **Table 2**).

**Table 1B:** Laboratory parameters and biomarkers.

Baseline characteristics of patients with IgAN treated with telitacicept (n=38)	
WBC,median (IQR), 10^6^/cm³	7.30(5.78-8.68)
Lymphocyte, median (IQR), 10^6^/cm³	2.09(1.57-2.50)
Urine protein, median (IQR), g/24h	1.75(1.16-3.19)
eGFR, mean(SD), ml/min/1.73m2	83.69(32.41)
Serum albumin, median (IQR), g/L	41.35(37.33-42.83)
Urinary RBC count, median(IQR), cells/hp	22.00(5.75-50.00)
Hgb, mean(SD), g/l	131.00(17.95)
BAFF, mean (SD), pg/ml(n=16)	270.48(94.18)
APRIL, mean (SD),ng/ml(n=16)	26.40(5.33)
Poly-IgA, mean (SD),ug/ml(n=16)	86.36(44.49)

IQR, interquartile range; SD, standard deviation; WBC, white blood cell; eGFR, estimated glomerular filtration rate; RBC, red blood cell; Hgb, hemoglobin; BAFF, b cell activating factor; APRIL, a proliferation-inducing ligand; Poly-IgA, poly-IgA immune complex.

#### 3.2.2 Survival analysis of remission rates

**Figure 3:**
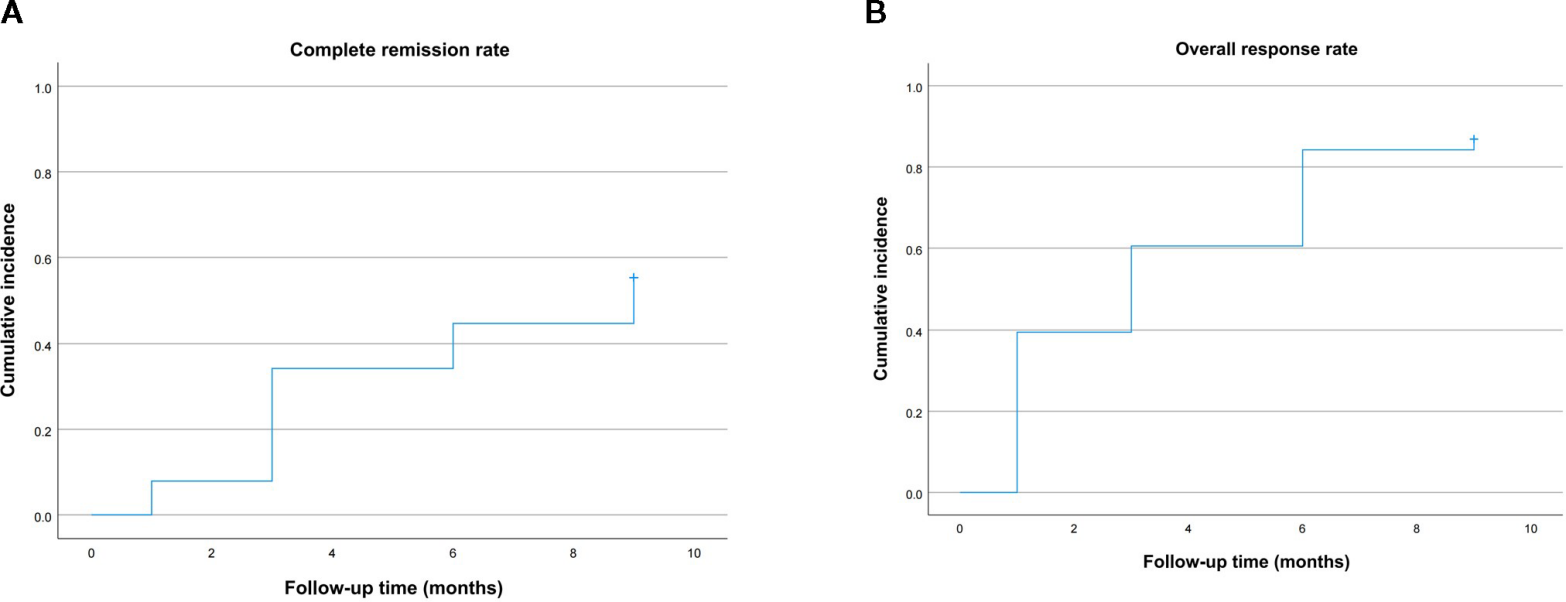
Kaplan-Meier curves of remission outcomes. **(A)** Cumulative incidence of complete remission in patients during telitacicept treatment follow-up; **(B)** Cumulative incidence of overall response in patients during telitacicept treatment follow-up.

Kaplan–Meier analysis was conducted to estimate the cumulative incidence of complete remission and overall remission during follow-up (**Figure 3**). The median time to complete remission was 9 months (95% CI: 4.49–13.51), and the median time to overall remission was 3 months (95% CI: 1.52–4.48). At the maximum follow-up time of 9 months, the complete remission rate reached 55.3% and the overall remission rate reached 86.8%.

### 3.3 Changes in laboratory parameters

#### 3.3.1 Changes in proteinuria, eGFR, and urinary RBC count

**Figure 4:**
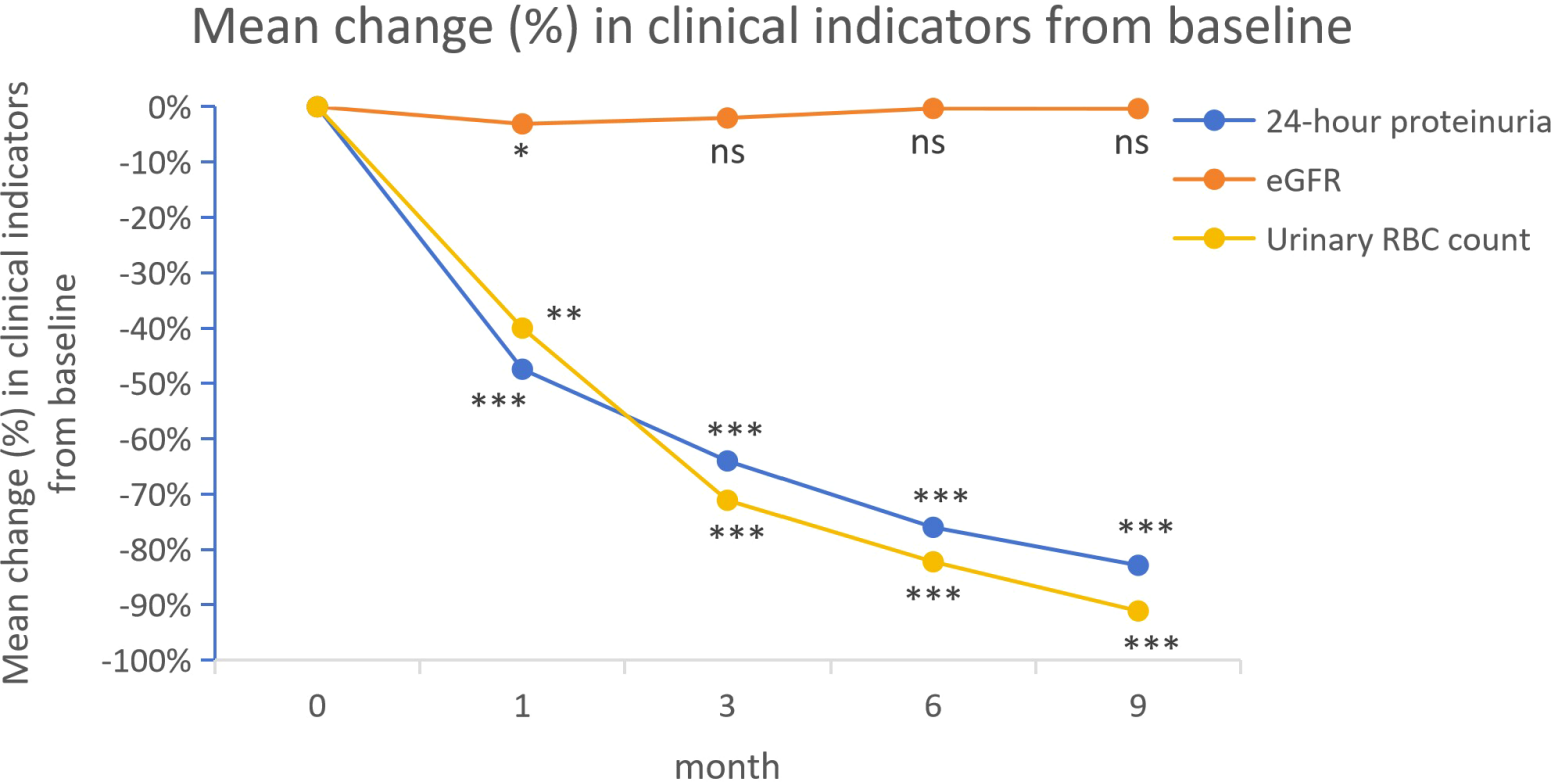
Percentage change from baseline in mean levels of 24-hour urinary protein, eGFR, and urinary RBC count (%) are plotted across visits. Statistical significance relative to baseline is indicated: *P<0.05, **P<0.01, ***P<0.001, ns, no significant difference.

At 6 months of telitacicept treatment, 24-hour urinary protein excretion decreased by 1.33 g/day (76.00%) from baseline (Z = –5.286, P < 0.001), urinary RBC count decreased by 18.5 cells/HPF (82.22%) (Z = –4.353, P < 0.001), and eGFR decreased by 0.26 ml/min/1.73 m² (0.31%, P = 0.891). By 9 months, urinary protein had decreased by 1.45 g/day (82.86%) (Z = –5.373, P < 0.001), urinary RBC count had decreased by 20.5 cells/HPF (91.11%) (Z = –5.331, P < 0.001), and eGFR had decreased by 0.29 ml/min/1.73 m² (0.35%, P = 0.893). Although a slight decrease in the eGFR was observed compared with baseline, it remained stable overall, with P > 0.05, indicating that this fluctuation is not clinically significant and renal function remains relatively stable. (**Figure 4**).

#### 3.3.2 Changes in Poly-IgA, BAFF, and APRIL levels

**Figure 5:**
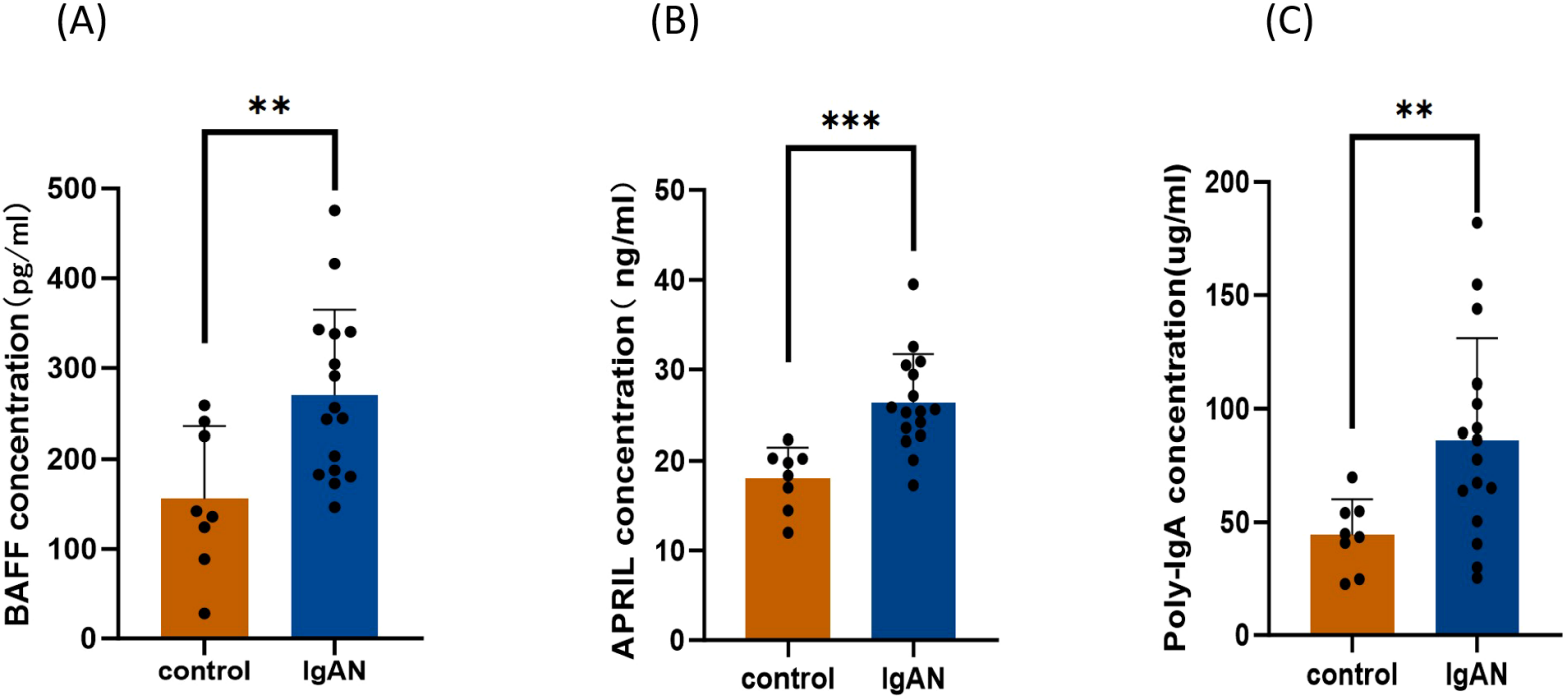
Comparison of biomarker concentrations between healthy control group and IgA nephropathy group: **(A)** BAFF, **(B)** APRIL, **(C)** Poly-IgA. **P<0.01, ***P<0.001. BAFF, b cell activating factor; APRIL, a proliferation-inducing ligand; Poly-IgA, poly-IgA immune complex.

Serum samples were obtained from 16 patients (tested subgroup) with continuously collected serum specimens; baseline characteristics comparison between these 16 patients and 22 untested patients showed no statistically significant differences in demographic, clinical, or laboratory parameters (see **Supplementary Table 1**). Samples from 8 healthy controls were also collected. The results showed that baseline levels of BAFF, APRIL, and Poly-IgA were significantly higher in patients with IgAN compared with healthy controls (P < 0.05). Specifically, BAFF levels were 270.48 ± 94.18 pg/ml versus 155.56 ± 80.39 pg/ml (P = 0.007) (**Figure 5A**), APRIL levels were 26.40 ± 5.33 ng/ml versus 18.05 ± 3.41 ng/ml (P < 0.001) (**Figure 5B**), and Poly-IgA levels were 86.36 ± 44.49 µg/ml versus 44.49 ± 15.68 µg/ml (P = 0.003) (**Figure 5C**).

**Figure 6:**
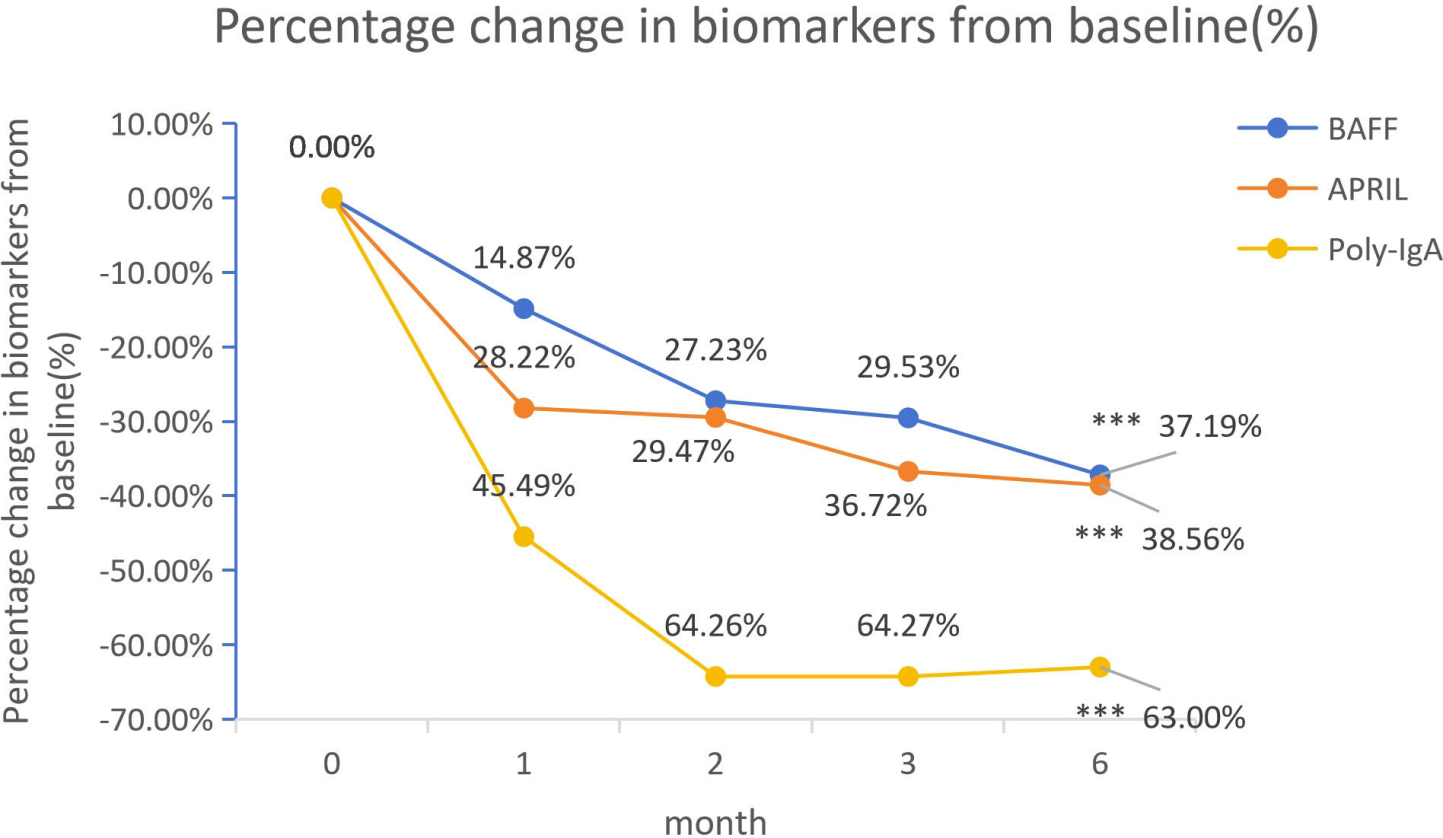
Percentage change from baseline in mean levels of BAFF, APRIL, and Poly-IgA are plotted across visits. Statistical significance relative to baseline at 6 months is indicated: ***P<0.001.

Longitudinal analysis of the 16 IgAN patients showed a consistent decline in all three biomarkers during telitacicept therapy. At 6 months, BAFF decreased by 100.59 pg/ml (37.19%) (t = 6.141, P < 0.001); APRIL decreased by 10.18 ng/ml (38.56%) (t = 9.927, P < 0.001); Poly-IgA decreased by 64.27% at 3 months (to 29.33 µg/ml) (Z = 3.516, P < 0.001) and by 63.00% at 6 months (to 30.37 µg/ml) (Z = 3.516, P < 0.001). The small increase in Poly-IgA at 6 months was not statistically significant (Z = 1.344, P = 0.179), indicating a stable and sustained downward trend overall (**Figure 6**).

### 3.4 Comparison between remission and non-remission groups

#### 3.4.1 Comparison of baseline characteristics

Among the 38 patients who completed the 9-month follow-up, 28 achieved overall remission and 10 did not. There were no significant differences between the remission and non-remission groups in age (37.86 ± 14.40 years vs. 39.80 ± 8.64 years, P = 0.618) or sex distribution (female: 42.9% vs. 40.0%, P = 0.999). According to the Oxford Classification, 39.3% of patients in the remission group were E1 and 67.9% were T1+T2, compared with 0% and 100% in the non-remission group (P < 0.05). The overall remission rate was significantly higher in E1 patients (100%, 11/11) than in E0 patients (63.0%, 17/27; P = 0.018), and similarly, the remission rate was significantly lower in T1+T2 patients (65.5%, 19/29) compared with T0 patients (100%, 9/9; P = 0.042). These findings suggest that patients with E1 lesions who received telitacicept combined with low-dose glucocorticoids tended to have a better therapeutic response and higher clinical remission rate compared with E0 patients. In contrast, the presence of tubular atrophy/interstitial fibrosis (T1+T2) may affect the overall long-term prognosis. Regarding eGFR, the remission group had a significantly higher eGFR (91.66 ± 31.49 ml/min/1.73 m²) than the non-remission group (61.37 ± 24.33 ml/min/1.73 m²; P = 0.009). No significant differences were observed between the two groups in other baseline clinical parameters, including 24-hour urinary protein, urinary RBC count, or serum albumin (all P > 0.05) (**Table 3**).

**Table 2:** Efficacy of Telitacicept combined with low-dose glucocorticoids in the treatment of IgA nephropathy.

Follow-up period	CR	PR	NR	OR
3 months	13(34.2%)	9(23.7%)	16(42.1%)	22(57.9%)
6 months	15(39.5%)	12(31.6%)	11(28.9%)	27(71.1%)
9 months	20(52.6%)	8(21.1%)	10(26.3%)	28(73.7%)

CR, Complete remission; PR, Partial remission; NR, No remission; OR, Overall remission.

#### 3.4.2 Predictive value of oxford classification

**Figure 7:**
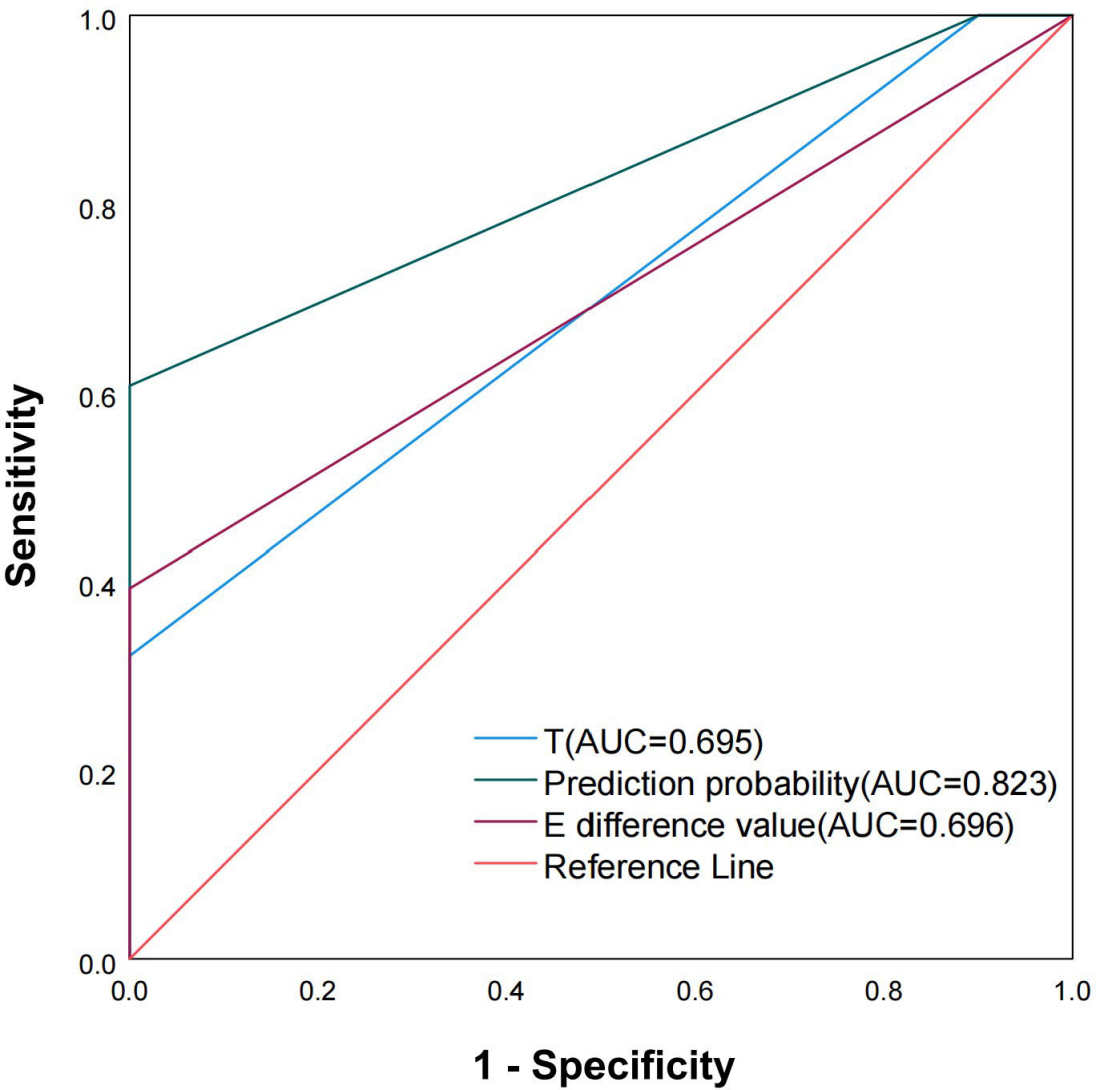
Receiver operating characteristic (ROC) curves for predicting complete remission. The ROC curves illustrate the predictive performance of different indicators for complete remission: T lesions (blue line, AUC = 0.695), E lesions (purple line, AUC = 0.696), and the combined predictive model integrating both E and T lesions (green line, AUC = 0.823). The red diagonal line represents the reference baseline (AUC = 0.5). AUC, area under the curve. E: endocapillary hypercellularity; T: tubular atrophy/interstitial fibrosis.

Univariate analysis results showed that both E lesions and T lesions are potential predictive factors. To evaluate the predictive performance of E lesions and T lesions in the Oxford classification for achieving overall remission after 9 months of treatment, we conducted ROC curve analysis for individual pathological indicators. The results showed that the AUC of T lesions for predicting remission was 0.695 (95% CI: 0.520–0.870, P > 0.05); the AUC of E lesions was 0.696 (95% CI: 0.529–0.863, P > 0.05). The AUC of both was slightly lower than the commonly used threshold of 0.70, and did not reach statistical significance. Considering the insufficient predictive ability of a single pathological indicator, we further constructed a binary Logistic regression comprehensive prediction model integrating E lesions and T lesions. This comprehensive model significantly improved the predictive performance, with the AUC increasing to 0.823, and the result was statistically significant (95% CI: 0.694–0.953, P < 0.05). The optimal cut-off value of this prediction model was 0.225, with a sensitivity of 60.7% and a specificity of 100% (**Figure 7**). In clinical translation, this model can serve as an effective risk early warning tool for the early identification of high-risk patients who are highly likely to be non-responsive to the telitacicept combination regimen, thereby providing an important basis for adjusting treatment strategies.

#### 3.4.3 Multivariate COX regression analysis of factors influencing clinical remission

**Figure 8:**
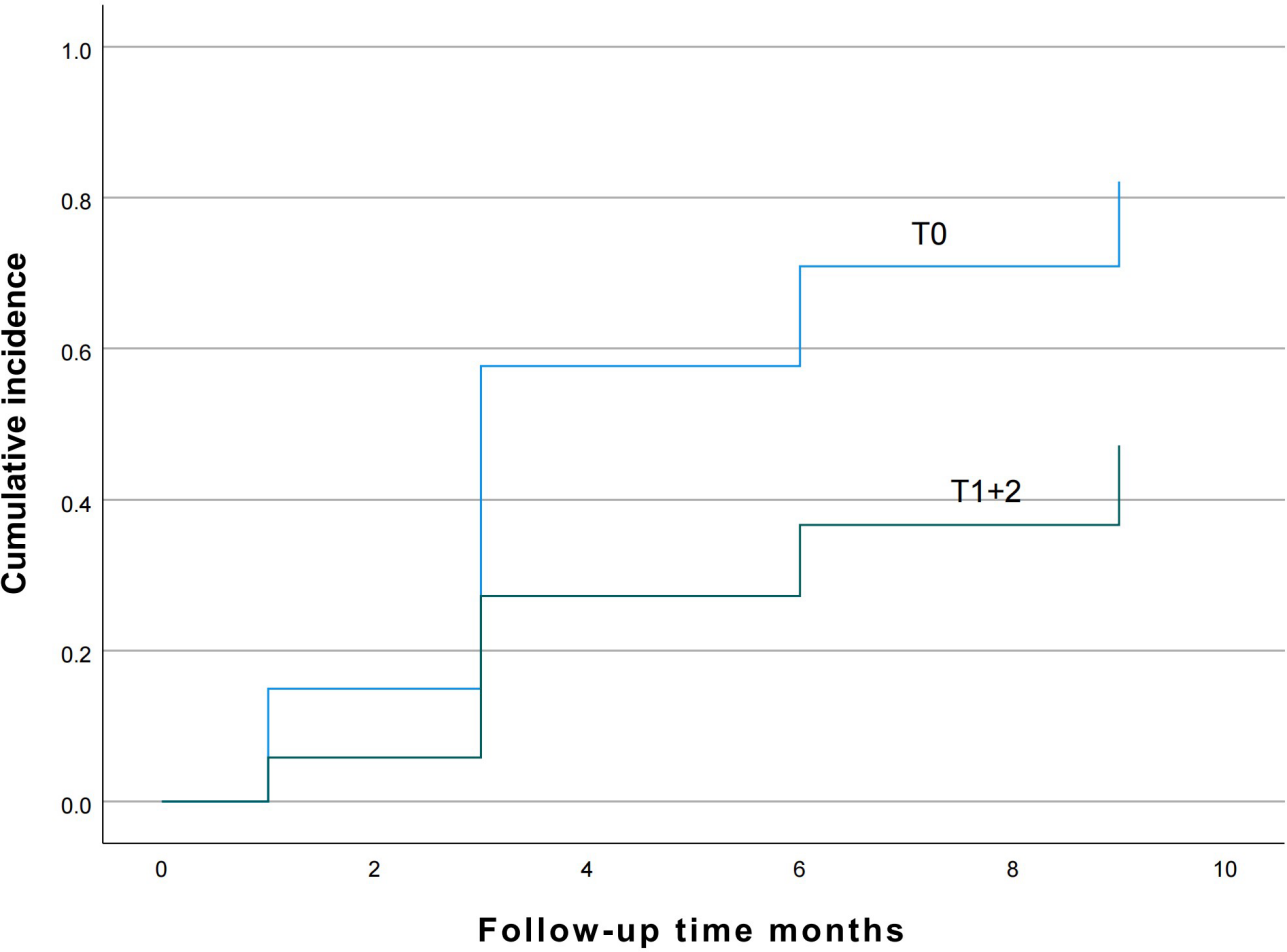
Adjusted survival curves of overall remission based on the multivariate Cox regression model, stratified by T lesion status. Kaplan-Meier curves showing the cumulative incidence of overall remission over follow-up time (months) in patients stratified by T lesion status. T0 = no tubular atrophy/interstitial fibrosis; T1 + 2 = presence of mild to severe tubular atrophy/interstitial fibrosis.

To evaluate the independent prognostic value of pathological and clinical indicators, a multivariate COX proportional hazards regression model was established. Based on the comparison of baseline characteristics between the remission and non-remission groups, the model included E and T lesions from the Oxford classification, as well as 24-hour urinary protein and eGFR from baseline clinical indicators. The model was overall statistically significant (likelihood ratio test χ² = 4.345, P = 0.037), indicating that the combination of these variables had a significant effect on predicting remission in IgAN. After adjusting for other factors in the model, tubular atrophy/interstitial fibrosis (T1+T2) was an independent risk factor for disease non-remission. Compared with the T0 group, the risk ratio of non-remission in the T1+T2 group was 2.70 (95% CI: 1.12–6.55; P = 0.028) (**Table 4**). Based on the results of the multivariate COX regression model, adjusted survival curves were plotted according to T lesions (**Figure 8**). The results showed that the median remission time of T0 patients (3 months, 95% CI: 1.05–4.95) was significantly shorter than that of T1+T2 patients. By the end of the 9-month follow-up, the Kaplan–Meier curve for complete remission in T1+T2 patients had not crossed 50%, so the median remission time was not reached in the T1+T2 group at the 9-month follow-up time point. At the maximum follow-up time of 9 months, the complete remission rate was 44.83%. The other three variables were not independent risk factors for disease non‑remission; their univariate Cox regression results are shown in **Supplementary Table 2**.

**Table 3:** Comparison of baseline characteristics between remission and non-remission groups.

Characteristics	Remission group (n=28)	Non-remission group (n=10)	*P* Value
Age, yr	37.86(14.40)	39.80(8.64)	0.618
Female sex, n (%)	12(42.9%)	4(40.0%)	0.999
Urine protein, g/24h	1.54(1.13-4.02)	2.24(1.46-2.87)	0.529
eGFR, ml/min/1.73m2	91.66(31.49)	61.37(24.33)	0.009
Serum albumin, g/L	40.35(34.75-42.48)	42.35(40.00-44.25)	0.115
Urinary RBC count, cells/hp	31.00(7.00-73.00)	17.50(4.75-24.75)	0.185
Hgb, g/l	129.61(17.30)	134.90(20.10)	0.431
Hypertension, n(%)	10(35.7%)	6(60.0%)	0.267
Diabetes, n(%)	0(0%)	1(10.0%)	0.263
Oxford classification, n			
M0/1	5/23	0/10	0.196
E0/1	17/11	10/0	0.018
S0/1	14/14	4/6	0.432
T0/1/2	9/19/0	0/9/1	0.042
C0/1/2	18/8/2	7/3/0	0.532

eGFR, estimated glomerular filtration rate; RBC, red blood cell; Hgb, hemoglobin; M, mesangial hypercellularity; E, endocapillary hypercellularity; S, segmental glomerulosclerosis; T, tubular atrophy/interstitial fibrosis; C, crescents.

#### 3.4.4 Serological indicators for assessing clinical remission

**Figure 9:**
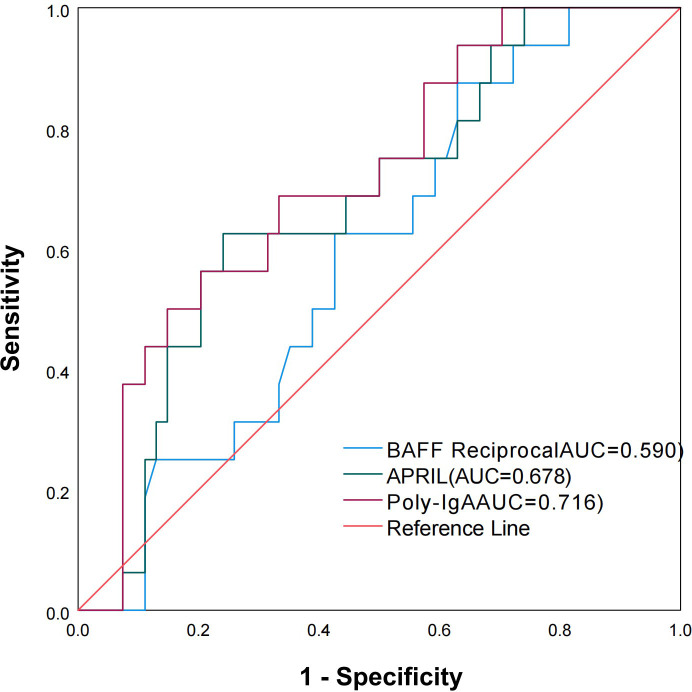
Receiver operating characteristic (ROC) curves of serum BAFF, APRIL, and Poly-IgA levels for evaluating clinical treatment response. BAFF: b cell activating factor (blue line, AUC = 0.590); APRIL, a proliferation-inducing ligand (green line, AUC = 0.678); Poly-IgA, poly-IgA immune complex (purple line, AUC = 0.716). The red diagonal line represents the reference line (AUC = 0.5). AUC, area under the curve.

ROC curve analysis was performed to determine the ability of BAFF, APRIL, and Poly-IgA to assess complete remission (CR) during treatment. Binary logistic regression analysis showed that all three biomarkers were significantly associated with CR (P < 0.05). Further ROC curve analysis revealed that BAFF had an AUC of 0.590 (95%CI:0.447-0.733, P = 0.278), indicating limited value in assessing clinical remission. APRIL showed moderate discriminatory ability with an AUC of 0.678 (95%CI:0.536-0.820, P = 0.031) and a cutoff value of ≤16.21 ng/ml, yielding 62.5% sensitivity and 75.9% specificity. Poly-IgA exhibited relatively strong assessment performance with an AUC of 0.716 (95%CI:0.580, 0.852, P = 0.009) and an optimal cutoff value of ≤28.51 µg/ml, corresponding to 56.3% sensitivity and 79.6% specificity (**Figure 9**, **Table 5**). This exploratory analysis suggests that lower serum Poly-IgA levels observed during treatment (e.g., ≤28.51 μg/ml) may be associated with clinical remission status. This preliminary finding needs to be validated in future prospective studies with rigorous design and sampling at fixed time points.

**Table 4:** Multivariate COX regression analysis of time to complete remission in patients.

Indicators	*P*	HR	95.0% CI
T	0.028	2.701	1.115-6.546
E	0.829	–	–
Urine protein	0.164	–	–
eGFR	0.154	–	–

T, tubular atrophy/interstitial fibrosis; E, endocapillary hypercellularity; eGFR, estimated glomerular filtration rate; HR, Risk ratio.

### 3.5 Correlation analysis between proteinuria and biomarkers

Serum APRIL and Poly-IgA levels showed significant positive correlations with 24-hour urinary protein levels at baseline and throughout follow-up (APRIL: R = 0.367, P = 0.002; Poly-IgA: R = 0.296, P = 0.013). In contrast, no meaningful correlation was observed between BAFF and urinary protein excretion (P = 0.878) (**Table 6**). Furthermore, serum concentrations of BAFF, APRIL, and Poly-IgA were positively correlated with one another (P < 0.01), indicating shared regulatory or pathogenic pathways (**Table 7**).

**Table 5:** Value of serum BAFF, APRIL, and Poly-IgA levels for evaluating clinical treatment response.

Biomakers	AUC	95%CI	cut-off value	sensitivity(%)	specificity(%)	*P* value
BAFF Reciprocal	0.590	0.447, 0.733	170.59	87.5	37.0	0.278
APRIL	0.678	0.536, 0.820	16.21	62.5	75.9	0.031^*^
Poly-IgA	0.716	0.580, 0.852	28.51	56.3	79.6	0.009^**^

BAFF, b cell activating factor; APRIL, a proliferation-inducing ligand; Poly-IgA, poly-IgA immune complex; P value: *P<0.05, **P<0.01.

**Table 6:** Correlation between 24-hour urine protein and biomarkers in Telitacicept groups.

Biomarkers	Spearman correlation coefficient	*P* value
BAFF	-0.019	0.878
APRIL	0.367	0.002
Poly-IgA	0.296	0.013

BAFF, b cell activating factor; APRIL, a proliferation-inducing ligand; Poly-IgA, poly-IgA immune complex.

### 3.6 Safety evaluation

No deaths, instances of renal failure requiring replacement therapy, or progression to end-stage renal disease were observed during the treatment period. As summarized in **Table 8**, adverse events (AEs) occurred in 5 patients (13.16%), including 4 cases of injection site reactions (10.53%) and 1 case of upper respiratory tract infection (2.63%). All AEs were mild to moderate in severity, and no serious adverse events were reported.

**Table 7:** Correlation between biomarkers in Telitacicept groups.

Biomakers	Correlation statistics	BAFF	APRIL	Poly-IgA
BAFF	Coefficient	-	0.462	0.318
	*P* value	-	<0.001	0.007
APRIL	Coefficient	0.462	-	0.310
	*P* value	<0.001	-	0.009

BAFF, b cell activating factor; APRIL, a proliferation-inducing ligand; Poly-IgA, poly-IgA immune complex.

**Table 8:** Incidence of adverse events during the 9-month treatment period.

Events, n (%)	Telitacicept 160 mg (n =38)
AE,n(%)	5(13.16%)
Serious AE,n(%)	0
AE resulting in end-stage renal disease,n(%)	0
Injection site reactions, n (%)	4(10.53%)
Upper respiratory tract infection, n (%)	1(2.63%)

## 4 Discussion

In this study, we first explored the efficacy and safety of telitacicept combined with low-dose glucocorticoids in the treatment of patients with IgA nephropathy (IgAN), and simultaneously evaluated the value of BAFF, APRIL, and Poly-IgA as serum biomarkers for assessing treatment response. Our results confirmed that telitacicept can significantly reduce proteinuria and protect renal function with a favorable safety profile. Notably, baseline levels of BAFF, APRIL, and Poly-IgA were significantly elevated in IgAN patients and decreased significantly after treatment, reflecting suppression of pathogenic B cell activation and downstream production of Gd-IgA1 and IgA immune complex. We also observed that among the biomarkers in the context of this study, Poly-IgA had the strongest correlation with clinical remission and the greatest discriminatory value in identifying responders. These findings are consistent with the mechanism of action of telitacicept, which reduces the production of pathogenic IgA and the formation of IgA immune complex by simultaneously blocking BLyS and APRIL signaling. In addition to confirming the relationship between efficacy and serum biomarkers, we identified that patients with T1/2 lesions according to the Oxford Classification had lower remission rates, indicating that underlying histopathological severity may influence treatment outcomes.

Previous clinical evidence from a phase II randomized controlled trial showed that after 24 weeks of treatment with 240 mg telitacicept, proteinuria was reduced by approximately 49%, and after treatment with 160 mg telitacicept, urinary protein decreased by 25% (13). Our observational data extended these observations by confirming the durability of response with a larger sample size and longer follow-up period, showing that proteinuria was reduced by 76.00% and 82.86% from baseline at 6 and 9 months of treatment, respectively, while eGFR remained stable throughout the treatment process. Notably, no serious adverse events were observed during the 9-month treatment period. The overall incidence of adverse events was low (13.16%), and all events were mild to moderate in severity, which is consistent with the favorable safety profile previously reported for telitacicept in the treatment of systemic lupus erythematosus (14) and rheumatoid arthritis (15). Furthermore, a multicenter cohort study by He et al. indicated that, compared to mycophenolate mofetil plus low-dose glucocorticoids, telitacicept combination therapy provided superior proteinuria reduction (−62.5% vs −52.9%) and a higher complete remission rate (33.3% vs 16.1%) at 12 months, along with renal function stabilization, reduced cumulative glucocorticoid exposure, and improved safety (24). Although the role of glucocorticoids in this study cannot be excluded, the TESTING study showed that treatment with low-dose methylprednisolone alone reduced the patients’ time-averaged urinary protein by 0.83 g/d compared with placebo (95% CI: 0.40–1.25). In contrast, the urinary protein-lowering effect achieved by the combination regimen of telitacicept with a smaller dose of glucocorticoid in this study showed a better trend than the previously reported hormone monotherapy (26). Moreover, the two may have a synergistic effect without increasing additional safety risks, providing a new reference for clinical treatment.

In the management of IgA nephropathy, preventing and reducing the formation of pathogenic IgA immune complex has become a key therapeutic goal. Recent therapeutic advances have focused on multiple pathogenic pathways, including the regulation of the APRIL and BAFF cytokine pathways. Studies have shown that at week 24 of treatment, plasma Gd-IgA1 levels decreased by 43.9% and Poly-IgA decreased by 41.3% with 160 mg telitacicept, and plasma Gd-IgA1 decreased by 50.4% and Poly-IgA decreased by 67.2% with 240 mg telitacicept (25). Our findings confirm these observations. Serum levels of BAFF, APRIL, and Poly-IgA were significantly higher in IgAN patients compared with healthy controls, which is consistent with the notion that aberrant B cell activation, excessive production of Gd-IgA1, and formation of IgA immune complex are key pathogenic steps in IgAN (5, 10, 11). Furthermore, telitacicept treatment resulted in significant reductions in all three biomarkers. The decreases in BAFF and APRIL reflect the drug’s mechanism as a TACI-Fc fusion protein that directly neutralizes these ligands (12). The pronounced decline in Poly-IgA (approximately 63% after 6 months) is likely an indirect downstream effect of BAFF/APRIL inhibition, leading to reduced differentiation or impaired survival of Gd-IgA1-producing plasma cells, thereby resulting in decreased formation of Poly-IgA (10). This observation is consistent with findings from atacicept trials, which demonstrated significant, dose-dependent reductions in Gd-IgA1 levels (16–19), collectively reinforcing the therapeutic value of targeting this upstream immunopathogenic pathway in IgAN.

Given the established pathogenic roles of Gd-IgA1 and Poly-IgA in IgAN, we further investigated whether serum concentrations of BAFF, APRIL, and Poly-IgA can be used to assess clinical treatment response. Therefore, we first performed binary logistic regression analysis, which showed that all three biomarkers were significantly associated with complete remission (P < 0.05). Further ROC curve analysis indicated that Poly-IgA (AUC = 0.716) had markedly greater discriminatory ability for identifying clinical remission than BAFF (AUC = 0.590) or APRIL (AUC = 0.678). This superiority is related to the fact that Poly-IgA is the main source of renal immune deposits and plays a key role in the four-hit model of IgAN pathogenesis (5, 20). Although BAFF and APRIL act as upstream regulators in the same pathogenic cascade, their circulating levels may be affected by broader immunologic processes, thereby reducing their specificity. In contrast, reductions in Poly-IgA more directly reflect inhibition of the core pathogenic pathway targeted by telitacicept. Despite the limited sample size, our analysis revealed that lower on−treatment Poly−IgA levels (e.g., ≤28.51 μg/ml, measured concurrently with the remission assessment) were significantly associated with a higher probability of clinical remission. This novel observation provides the first clinical evidence linking Poly-IgA dynamics to telitacicept response, generating a testable hypothesis for future validation in larger, prospective studies with standardized sampling timepoints.

Our subgroup analysis showed that patients with E1 lesions tended to have a better therapeutic response and higher clinical remission rate after receiving telitacicept combined with low-dose glucocorticoids compared with E0 patients. In contrast, the presence of tubular atrophy/interstitial fibrosis (T1+T2) may significantly reduce the remission rate. To clarify the predictive value of E and T lesions for clinical remission, we conducted ROC curve analysis. In this cohort, the AUC values for E lesions alone or T lesions alone (0.695, 0.696) were slightly below the 0.70 threshold and did not reach statistical significance. However, the model combining E and T achieved a higher AUC (0.823). This suggests that in clinical practice, a more refined integrated interpretation of the Oxford classification, rather than viewing individual lesions in isolation, may provide useful information for risk stratification. Nevertheless, it must be emphasized that the sample size of our study is small, and this pathological model needs to be validated in prospective large-sample cohorts.

To exclude confounding effects caused by other indicators, we performed a multivariate COX regression risk model, which showed that only T lesions remained significant after strict adjustment, establishing it as an independent prognostic factor affecting efficacy (HR = 2.70, 95% CI: 1.12–6.55; P = 0.028). This finding is consistent with a retrospective study of 101 IgAN patients, whose survival analysis showed that patients with M or T lesions receiving glucocorticoids combined with mycophenolate mofetil had significantly reduced renal survival (21). In addition, Tesar et al. reported that the rate of eGFR decline was significantly faster in patients with T1 compared with T0 lesions, regardless of whether they received renin–angiotensin system blockers (RASi) alone or in combination with corticosteroids (22). These results align with the KDIGO guideline, which emphasizes that the extent of chronic fibrotic injury is among the strongest predictors of IgAN prognosis (4). Variables such as E lesions, 24-hour urinary protein, and eGFR lost significance in the multivariate model, revealing that their associations may be due to confounding factors. Collectively, our findings suggest that the therapeutic efficacy of telitacicept is closely linked to baseline pathological severity, with patients exhibiting T1/2 lesions deriving less benefit compared with those presenting with T0 pathology.

This study has several limitations. First, as a single-center retrospective analysis, the sample size is relatively small, making it impossible to conduct a longitudinal analysis at fixed time points. Therefore, we performed an exploratory cross-sectional analysis to evaluate the association between biomarker levels at any time point during treatment and the concurrent remission status. Hence, the results of the biomarker analysis in this study are only used to generate hypotheses and need to be further verified in future prospective studies. Second, the lack of a control group receiving conventional immunosuppressive therapy limits direct comparison with established treatment regimens, and the effect of glucocorticoids on treatment cannot be excluded. It is recommended that future randomized controlled trials be designed to compare telitacicept combined with glucocorticoids versus glucocorticoids alone. Third, although the dose of telitacicept 160 mg/week used was in line with clinical practice at that time, it was not the optimal dose (240 mg/week) verified by RCTs. This may have limited the observed magnitude of efficacy and increased uncertainty in the interpretation of results. However, it also provides early real-world data reference for future confirmatory RCTs verifying higher doses (such as 240 mg). Finally, verification bias in this study may lead to an overestimation of the efficacy evaluation of biomarkers. It is suggested that future studies should adopt consecutive enrollment and a predefined sample collection framework to avoid such bias.

In conclusion, this observational study demonstrates that telitacicept effectively reduces proteinuria in patients with IgAN and exhibits a favorable safety profile. Dynamic monitoring of serum Poly-IgA appears to be a promising and practical approach for assessing treatment response. Future large-scale, multicenter randomized controlled trials are needed to further define the role of telitacicept within the IgAN treatment landscape and to validate personalized therapeutic strategies informed by biomarker profiles and pathological classification.

## Data Availability

The original contributions presented in the study are included in the article/**Supplementary Material**. Further inquiries can be directed to the corresponding author.

## Supplementary material

Supplementary Table 1 Comparison of baseline characteristics between the tested group and the untested group.

Supplementary Table 2 Univariate COX regression analysis of time to complete remission in patients.
