# Supplementary material for: Poly-IgA immune complex as a monitoring biomarker of response to telitacicept in IgA nephropathy
Source: Front Immunol. 2026 May 29;17:1751549. doi: 10.3389/fimmu.2026.1751549 (PMC13260420; doi:10.3389/fimmu.2026.1751549)
Supplement: Supplementary Table 1 — Comparison of baseline characteristics between the tested group and the untested group. [file SupplementaryFile1.pdf]

Supplementary Table 1. Comparison of baseline characteristics between the tested group and the untested group

	detection group（n=16)	undetected group(n=22)	P Value
Age，yr	39.50（14.98）	37.55（11.74）	0.655
Female sex, n (%)	9（56.3%）	7（31.8%）	0.188
Urine protein, g/24h	2.01（1.24-3.83）	1.54（1.13-2.51）	0.564
eGFR，ml/min/1.73m2	85.70（42.18-122.78）	84.11（59.13-101.00）	0.767
Serum albumin，g/L	41.00（38.58-43.28）	41.40（36.40-43.08）	0.988
Urinary RBC count, cells/ hp	14.50（6.25-40.50）	33.00（5.75-73.00）	0.178
Hgb , g/l	127.50（15.77）	133.55（19.35）	0.312
Hypertension, n(%)	8（50.0%）	8（36.4%）	0.511
Diabetes, n(%)	0（0%）	1（4.5%）	0.999
Oxford classification, n			
M0/1	1/15	4/18	0.374
E0/1	12/4	15/7	0.729
S0/1	6/10	12/10	0.342
T0/1/2	5/10/1	4/18/0	0.450
C0/1/2	9/6/1	16/5/1	0.323

eGFR: estimated glomerular filtration rate; RBC: red blood cell; Hgb: hemoglobin; M: mesangial hypercellularity; E: endocapillary hypercellularity; S: segmental glomerulosclerosis; T: tubular atrophy/interstitial fibrosis; C: crescents;

Supplementary Table 2. Univariate COX Regression Analysis of Time to Complete Remission in Patients

	P	HR	95.0% CI
E	0.602	0.785	0.316-1.950
Urine protein	0.456	0.907	0.703-1.171
eGFR	0.047	1.013	0.999-1.027

E: endocapillary hypercellularity; eGFR: estimated glomerular filtration rate; HR: Risk ratio
